# Multiple-Factor Analyses of Futile Recanalization in Acute Ischemic Stroke Patients Treated With Mechanical Thrombectomy

**DOI:** 10.3389/fneur.2021.704088

**Published:** 2021-08-19

**Authors:** Hui Pan, Changchun Lin, Lina Chen, Yuan Qiao, Peisheng Huang, Bin Liu, Yueqi Zhu, Jingjing Su, Jianren Liu

**Affiliations:** ^1^Department of Neurology, Shanghai Ninth People's Hospital, Shanghai Jiao Tong University School of Medicine, Shanghai, China; ^2^Department of Neurology, Shanghai Minhang Hospital, Fudan University, Shanghai, China; ^3^Department of Interventional Radiology, Shanghai Sixth People's Hospital, Shanghai Jiao Tong University School of Medicine, Shanghai, China

**Keywords:** acute ischemic stroke, futile recanalization, mechanical thrombectomy, NIHSS score, collateral circulation

## Abstract

**Background and Purpose:** Acute ischemic stroke (AIS) is a serious threat to the life and health of middle-aged and elderly people. Mechanical thrombectomy offers the advantages of rapid recanalization, but the response of patients to this treatment varies greatly. This study investigated the risk factors for futile recanalization in AIS patients after thrombectomy through multivariate analyses.

**Methods:** A retrospective study was conducted in AIS patients with anterior circulation occlusion from a derivation cohort and a validation cohort who underwent thrombectomy and reperfusion defined as a modified Thrombolysis in Cerebral Infarction (mTICI) score of 2b/3. Using the modified Rankin Scale (mRS) at 90 days after the operation, the patients were divided into two groups, the meaningful recanalization group (mRS ≤ 2), and the futile recanalization group (mRS > 2). Multivariate logistic regression analyses were performed, and the receiver operating characteristic (ROC) curve was used to construct a risk prediction model for futile recanalization. The performance of prediction model was evaluated on the validation cohort.

**Results:** A total of 140 patients in the derivation cohort were enrolled, 46 patients in the meaningful recanalization group and 94 patients in the futile recanalization group. The two groups were significantly different in age, preoperative National Institute of Health Stroke Scale (NIHSS) score, and collateral circulation ASITN/SIR grade (*P* < 0.05). In multivariate regression analyses, patients' age ≥ 71, NIHSS ≥ 12, and ASITN/SIR ≤ 3 were risk factors for futile recanalization. Hence, an ANA (Age-NIHSS-ASITN/SIR) score scale consisting of age, NIHSS score, and ASITN/SIR grade factors can effectively predict the risk for futile recanalization (area under curve 0.75, 95% CI 0.67–0.83, specificity 67.4%, and sensitivity 73.4%). The proportion of patients with futile recanalization in ANA groups 0, 1, 2, and 3 were 21.05, 56.76, 79.03, and 90.91%, respectively. Furthermore, ANA score scale had also a good performance for predicting futile recanalization on the validation cohort.

**Conclusions:** Old age, high baseline NIHSS, and poor collateral circulation are risk factors for futile recanalization in AIS patients treated with thrombectomy. An ANA score that considers age, NIHSS, and collateral ASITN/SIR can effectively predict the risk for futile recanalization. Further studies with a larger sample size are needed to validate the prognostic value of this combined score for futile recanalization.

## Introduction

Acute ischemic stroke (AIS) has become a serious disease burden because of its high mortality and disability (1). The key in treating AIS patients is to reopen the occluded arteries as early as possible. With the technological development of intravascular interventional therapy and the update of clinical guidelines, intravascular mechanical thrombectomy therapy has become widely accepted by clinicians (2, 3). However, futile recanalization after mechanical thrombectomy is common in AIS patients (4, 5). Futile recanalization is defined as poor clinical outcome, or treatment failure despite adequate vessel recanalization, following endovascular treatment in AIS patients (4). It has been reported that futile recanalization was observed in almost one half of patients after endovascular treatment and the rate of futile recanalization was 40.5–54.5% (6–9). It may be related to poor collateral circulation, subacute re-occlusion, massive hypoperfusion, and/or damage to capillaries and brain autoregulation.

Previous studies have suggested that certain imaging parameters or neural function evaluation methods can be used to predict futile recanalization and identify patients who may not be suitable for endovascular treatment (6, 10). Advanced age, severer neurological deficits assessed by the baseline National Institute of Health Stroke Scale (NIHSS), and female gender are considered to be the independent predictors of futile recanalization (7, 11). Delayed endovascular treatment and use of general anesthesia have been proposed as the important determinants of unfavorable outcomes in those patients with recanalization (8, 12). Baseline Alberta Stroke Program Early Computed Tomography Score (ASPECTS) from the non-contrast CT (NCCT) and CT angiography source images (CTA-SI) are useful in predicting futile recanalization and could be valuable tools for treatment decisions regarding revascularization therapies (13). Similarly, studies employing NCCT to determine the risk factors in AIS patients treated with mechanical thrombectomy have revealed that the presence of leukoaraiosis and brain atrophy is associated with poor outcome in recanalized patients (14, 15). Furthermore, another study applying diffusion-weighted magnetic resonance imaging (DWI) to predict futile recanalization has demonstrated that patients with large preintervention DWI lesions in the deep white matter may be poor candidates for endovascular therapy (6). Therefore, the use of functional imaging such as CT and magnetic resonance imaging (MRI) solely, or in combination, provides encouraging results as independent prognostic predictors for futile recanalization (16, 17). However, adopting these approaches during clinical practice may not be feasible; it remains unclear due to various issues among studies, including a small number of patients enrolled, and large variation in clinical characteristics and treatment strategies (18).

Studies on risk factors for futile recanalization in the Chinese AIS cohort are scarce. A substantial medical resource is required to identify underlying risks during mechanical thrombectomy treatment. We analyzed the risk factors for futile recanalization in a Chinese AIS cohort after mechanical thrombectomy. We also evaluated and validated whether the combination of these factors would improve prognostic value for the prediction of futile recanalization in such patients.

## Materials and Methods

### Study Design and Patients

This study was a retrospective study and included 2 cohorts of AIS patients from January 2016 to July 2020. A derivation cohort of 140 patients (85 males and 55 females) were admitted at the Department of Neurology, Shanghai Ninth People's Hospital, Shanghai Jiao Tong University School of Medicine. A validation cohort of 154 patients (98 males and 56 females) were from the Shanghai Minhang Hospital and Shanghai Sixth People's Hospital. All the patients had undergone mechanical thrombectomy treatment and recanalization in this study. Post-intravascular therapy reperfusion was graded using the modified Thrombolysis in Cerebral Infarction (mTICI) scales. The mTICI scores of 2b/3 were considered recanalization after mechanical thrombectomy (19). The mTICI grade was retrospectively confirmed by 2 neuroradiologists blinded to clinical and procedural data. According to the modified Rankin Scale (mRS) score at 90 days after the operation, the patients were divided into the meaningful recanalization group (mRS ≤ 2 points, good prognosis) and the futile recanalization group (mRS > 2 points, poor prognosis) (4, 20).

Further inclusion criteria were as follows: acute anterior large vessel occlusion as assessed via CTA, baseline ASPECTS ≥ 6 points (21), degree of preoperative neurological deficits assessed by the NIHSS score ≥ 2 points (22), and mechanical thrombectomy performed within 6 h of symptom onset without contraindications, or a small infarct core, and moderate-to-good collateral circulation on CT and CTA, or clinical symptoms mismatched with imaging findings (i.e., preoperative NIHSS score showing significant neurological deficits but small infarct core area in DWI or CT perfusion) within 6–24 h of symptom onset (17, 23, 24), plus digital subtraction angiography (DSA) showing good collateral circulation. Patients were excluded from the study if they had severe heart, kidney, or liver failure. AIS patients suffering from vasculitis or coagulation disorders, and subjects diagnosed with intracerebral hemorrhage were also excluded. The exclusion criteria for intravenous alteplase thrombolysis were as follows: delayed arrival time > 4.5 h after stroke onset, previous intracranial hemorrhage, stroke or brain trauma within 3 months, systolic blood pressure of more than 180 mmHg, platelet <100,000/μL, glucose level <2.7 mmol/L, active Partial Thromboplastin Time (aPTT) > 40 s, International Normalized Ratio (INR) > 1.7, and patient/family refusal (25).

This study was approved by the institutional ethics committee (SH9H-2020-T390-2) and informed consent forms were signed based on the study design.

### Clinical Assessments

Clinical data regarding sex, age, history of stroke or transient ischemic attack (TIA), and vascular disease risk factors (e.g., smoking, drinking, hypertension, diabetes, coronary heart disease, and atrial fibrillation) were recorded. Stroke subtype was based on the Trial of ORG 10172 in Acute Stroke Treatment (TOAST) classification, such as large-artery atherosclerosis (LAA), cardioembolism (CE), stroke of other determined cause (ODC), and stroke of undetermined etiology (SUE). Neurological deficits were evaluated using the preoperative NIHSS as the baseline for disability assessment. Imaging parameters such as the baseline ASPECTS based on CT scans, the American Society of Intervention and Therapeutic Neuroradiology/Society of Interventional Radiology (ASITN/SIR) grade of collateral circulation based on DSA, the arterial occlusive lesion (AOL) grade for recanalization after intravascular therapy and the modified Fazekas scale for leukoaraiosis were also recorded. All imaging data were assessed by 2 experienced neuroradiologists blinded to the clinical information.

### Endovascular Treatment

The patients were immediately transported to the neuroradiography suite. They received local or general anesthesia with endotracheal intubation at the discretion of the anesthesiologists. Endovascular treatment was performed by 2 experienced neurointerventionalists. Briefly, the patient's femoral artery was punctured and DSA was performed to determine the occlusion site. Using coaxial catheter technology, the tip of micro-catheter (Rebar 21/27, EV3, USA) was put at the distal end of the occluded artery under the guidance of a micro-guide wire (0.014 in Synchro-14, Stryker, U.S.), then the micro-guide wire was withdrawn. Subsequently, the whole cerebral angiography confirmed that the micro-catheter was in the arterial lumen. The Solitaire^TM^ AB embolization stents (EV3, USA) with diameters of 4–6 mm and lengths of 15–30 mm were selected according to the diameter of the occluded blood vessels. The stent was introduced to the distal end of the occlusion through the micro-catheter and then was released. Then the contrast agents for visualization were injected, and negative pressure was applied to the catheter to slowly withdraw the stent and remove the thrombus. Recanalization of the main arteries and branches was confirmed by re-examination, indicating that the thrombus was successfully removed. The number of stent retriever passes per procedure was generally no more than 4 times. In the event that residual stenosis > 50% remained after the procedure, either balloon dilation and/or stent implantation angioplasty were performed after comprehensive consideration. At the end of the procedure, the patients were transported to the neurological intensive care unit for standard stroke care in the charge of neurologists.

### Statistical Analyses

R software (version number 3.6.2; packages table 1, tidyverse, pROC and rms) and SPSS software (version number 22.0; IBM company) were used for statistical analyses. Quantitative data were described as medians and interquartile ranges (IQR) and categorical variables were described as numbers and percentages of cases. Analyses of the quantitative differences between two groups were compared using the Mann-Whitney U test. Chi-square or Fisher's exact tests were used in statistical analyses of categorical variables. The test-retest reliability was assessed by calculating Intraclass Correlation Coefficients (ICC) for the imaging parameters. An ICC between 0.6 and 0.8, and higher was regarded as good and excellent, respectively (26). Using futile recanalization as the dependent variable, general clinical data (sex, age), neurological deficits assessment (NIHSS), and functional imaging data (ASPECTS, ASITN/SIR and AOL grades) were used as independent variables. Briefly, univariate logistic regression analyses were used to identify risk factors for futile recanalization, and the odds ratio (OR) and 95% confidence interval (CI) were described. Subsequently, multivariate logistic regression analyses were used to identify independent risk factors for futile recanalization after adjustment of potential confounders (*P* < 0.05). To evaluate prediction values of variables solely, or in combination, receiver operating characteristic (ROC) curves were plotted. The OR and 95% CI were calculated. *P*-value < 0.05 was considered statistically significant.

## Results

### Comparison of Clinical Characteristics in the Study Population

In all, 140 AIS patients in the derivation cohort with anterior circulation occlusion who were recanalized after mechanical thrombectomy and evaluated by postoperative mTICI with scores of 2b/3 were included in the analyses (19). The clinical data of all the patients were presented in Table 1. For the continuous variables reported as median values, the numbers in round brackets were IQR. The postoperative 90-day mRS was used to assess neurological function as an indicator of the effects of endovascular treatment (4, 20). The subjects were divided into two groups: meaningful recanalization group (mRS ≤ 2 points, good prognosis) and futile recanalization group (mRS > 2, poor prognosis) (Table 1). Overall, 94 patients (67.1%) were in the futile recanalization group, of which 54 were male (57.4%, *P* > 0.05 compared to the meaningful recanalization group), with a median age of 71.0 (62, 80) years (*P* = 0.004 compared to the meaningful recanalization group), 46 patients (48.9%, *P* = 0.017) with atrial fibrillation, and baseline NIHSS score of 16 (12, 20) (*P* < 0.001). There was also a statistical difference in the ASITN/SIR grade of DSA collateral circulation between the two groups (*P* < 0.001). As shown in Table 1, there were no significant differences in other clinical and imaging features, including smoking, drinking, hypertension, diabetes, coronary heart disease, stroke or TIA history, TOAST classification, baseline blood pressure, ASPECTS, and AOL classification (*P* > 0.05). The ICC values for the imaging parameters were reported in Table 2, which were acceptable and ranged from ICC = 0.63–0.96.

**Table 1:** Comparison of clinical characteristics between meaningful recanalization and futile recanalization groups after mechanical thrombectomy in AIS patients.

**Characteristics**	**Total (*n* = 140)**	**Meaningful recanalization (mRS ≤ 2, *n* = 46)**	**Futile recanalization (mRS > 2, *n* = 94)**	***P-*values**
Sex (male), *n* (%)	85 (60.7%)	31 (67.4%)	54 (57.4%)	0.343
Age, year, median (IQR)	67.5 (59–78)	64.0 (55–72)	71.0 (62–80)	0.004
Smoking, *n* (%)	42 (30.0%)	17 (37.0%)	25 (26.6%)	0.289
Drinking, *n* (%)	26 (18.6%)	11 (23.9%)	15 (16.0%)	0.365
Hypertension, *n* (%)	83 (59.3%)	24 (52.2%)	59 (62.8%)	0.310
Diabetes, *n* (%)	37 (26.4%)	11 (23.9%)	26 (27.7%)	0.789
History of stroke or TIA, *n* (%)	21 (15.0%)	7 (15.2%)	14 (14.9%)	1.000
Coronary heart disease, *n* (%)	21 (15.0%)	7 (15.2%)	14 (14.9%)	1.000
Atrial fibrillation, *n* (%)	58 (41.4%)	12 (26.1%)	46 (48.9%)	0.017
TOAST, *n* (%)				0.323
LAA	84 (60.0%)	32 (69.6%)	52 (55.3%)	
CE	40 (28.6%)	9 (19.6%)	31 (33.0%)	
ODC	1 (0.7%)	0 (0.0%)	1 (1.1%)	
SUE	15 (10.7%)	5 (10.9%)	10 (10.6%)	
Baseline SBP, mmHg, median (IQR)	150 (132–166)	145 (127–160)	150 (136–172)	0.154
Baseline DBP, mmHg, median (IQR)	80 (73–94)	81 (78–94)	80 (71–93)	0.544
Glucose, mmol/L, median (IQR)	7.9 (6.4–9.2)	7.6 (6.9–8.9)	8.0 (6.3–9.3)	0.811
LDL-C, mmol/L, median (IQR)	2.89 (2.24–3.51)	3.23 (2.35–3.62)	2.62 (2.21–3.42)	0.176
Baseline NIHSS, median (IQR)	14 (10-19)	11 (8-15)	16 (12-20)	<0.001
Baseline ASPECTS, median (IQR)	9 (8-10)	9 (8-9)	8 (8-10)	0.381
DSA ASITN/SIR grade, median (IQR)	3 (2-3)	3 (2-4)	2 (2-3)	<0.001
**Baseline AOL grade**, ***n*****(%)**				
0	97 (69.3%)	28 (60.9%)	69 (73.4%)	0.244
1	22 (15.7%)	11 (23.9%)	11 (11.7%)	
2	20 (14.3%)	7 (15.2%)	13 (13.8%)	
3	1 (0.7%)	0 (0.0%)	1 (1.1%)	
Fazekas scale, median (IQR)	0 (0–0.5)	0 (0–1)	0 (0–0.5)	0.735
**Occlusion site**, ***n*****(%)**				
Middle cerebral artery	109 (80.1%)	35 (81.4%)	74 (79.6%)	0.804
Internal carotid artery	44 (32.4%)	19 (44.2%)	25 (26.9%)	0.045
Intravenous thrombolysis, *n* (%)	51 (36.4%)	15 (32.6%)	36 (38.3%)	0.638
Hemorrhage transformation, *n* (%)	50 (35.7%)	12 (26.1%)	38 (40.4%)	0.129
Number of passes per procedure, median (IQR)	1 (1-6)	1 (1-5)	1 (1-6)	0.362
Later than 6 h from onset to puncture, *n* (%)	52 (38.0%)	20 (47.6%)	32 (33.7%)	0.121
Time from onset to puncture, hour, median (IQR)	5.5 (4.0–7.5)	5.7 (4.2–8.3)	5.3 (3.9–7.4)	0.253
Time from arrival to puncture, hour, median (IQR)	2.4 (1.7–3.3)	2.7 (2.1–3.8)	2.3 (1.5–3.2)	0.038
Time from onset to recanalization, hour, median (IQR)	7.4 (5.7–9.1)	7.6 (6.0–9.7)	7.0 (5.7–9.0)	0.391
Procedure time, hour, median (IQR)	1.7 (1.3–2.3)	1.5 (1.2–2.3)	1.8 (1.3–2.3)	0.288

*AIS, acute ischemic stroke; mRS, modified Rankin Scale; IQR, interquartile range; TIA, transient ischemic attack; TOAST, Trial of Org 10172 in Acute Stroke Treatment; LAA, large-artery atherosclerosis; CE, cardioembolism; ODC, stroke of other determined cause; SUE, stroke of undetermined etiology; SBP, systolic blood pressure; DBP, diastolic blood pressure; LDL-C, low density lipoprotein cholesterol; NIHSS, National Institute of Health Stroke Scale; ASPECTS, Alberta Stroke Program Early CT Score; DSA, digital subtraction angiography; ASITN/SIR, American Society of Intervention and Therapeutic Neuroradiology/Society of Interventional Radiology; AOL, arterial occlusive lesion*.

**Table 2:** Test-retest intraclass correlation coefficients and 95% confidence intervals for the imaging parameters.

	**ICC (95% CI)**	***P-*values**
ASPECTS	0.81 (0.69–0.88)	0.000
ASITN/SIR grade	0.73 (0.65–0.80)	0.000
AOL grade	0.88 (0.84–0.92)	0.000
mTICI grade	0.63 (0.52–0.72)	0.000
Fazekas scale	0.96 (0.95–0.97)	0.000

*ICC, Intraclass Correlation Coefficients; CI, confidence interval; ASPECTS, Alberta Stroke Program Early CT Score; ASITN/SIR, American Society of Intervention and Therapeutic Neuroradiology/Society of Interventional Radiology; AOL, arterial occlusive lesion; mTICI, modified Thrombolysis in Cerebral Infarction*.

### Risk Factors for Futile Recanalization in AIS Patients Treated With Mechanical Thrombectomy

To identify the risk factors for futile recanalization in AIS patients after mechanical thrombectomy, we conducted univariate logistic regression analyses. As shown in Table 3, patient age (OR: 2.36, 95% CI: 1.35–4.13, *P* = 0.003), atrial fibrillation (OR: 2.72, 95% CI: 1.25–5.88, *P* = 0.011) and high baseline NIHSS score (OR: 2.22, 95% CI: 1.31–3.75, *P* = 0.003) were independent risk factors, while high ASITN/SIR grade (OR: 0.35, 95% CI: 0.21–0.57, *P* < 0.001) was a protective factor for futile recanalization. After further adjustment of confounders (including sex, age, smoking, drinking, hypertension, diabetes, history of stroke or TIA, coronary heart disease, atrial fibrillation, and TOAST) in multivariate logistic regression analyses, we found that advanced age (OR: 2.14, 95% CI: 1.09–4.20, *P* = 0.028) and high baseline NIHSS score (OR: 2.25, 95% CI: 1.29–3.94, *P* = 0.005) were still independent risk factors, while high ASITN/SIR grade (OR: 0.34, 95% CI: 0.20–0.58, *P* < 0.001) was still an independent protective factor for futile recanalization (Table 3).

**Table 3:** Univariate and multivariate logistic regression analyses of risk factors for futile recanalization after mechanical thrombectomy in AIS patients.

	**Univariate**		Multivariate^*^	
	**Crude OR (95% CI)**	***P-*values**	**Adjusted OR (95% CI)**	***P-*values**
Sex	1.53 (0.73–3.21)	0.259		
Age	2.36 (1.35–4.13)	0.003	2.14 (1.09–4.20)	0.028
Smoking	0.62 (0.29–1.31)	0.211		
Drinking	0.60 (0.25–1.45)	0.258		
Hypertension	0.65 (0.32–1.32)	0.232		
Diabetes	1.22 (0.54–2.75)	0.637		
History of stroke or TIA	0.97 (0.36–2.61)	0.960		
Coronary heart disease	0.97 (0.36–2.61)	0.960		
Atrial fibrillation	2.72 (1.25–5.88)	0.011	2.54 (0.93–6.92)	0.069
Baseline SBP	1.43 (0.89–2.28)	0.141		
Baseline DBP	0.93 (0.58–1.48)	0.754		
Glucose	1.07 (0.74–1.56)	0.714		
LDL-C	0.74 (0.46–1.20)	0.229		
Baseline NIHSS	2.22 (1.31–3.75)	0.003	2.25 (1.29–3.94)	0.005
Baseline ASPECTS	0.69 (0.38–1.25)	0.221		
DSA ASITN/SIR grade	0.35 (0.21–0.57)	<0.001	0.34 (0.20–0.58)	<0.001
Baseline AOL grade	0.82 (0.52–1.29)	0.390		
Intravenous thrombolysis	1.28 (0.61–2.70)	0.512		
Hemorrhage transformation	1.99 (0.92–4.34)	0.807		
Time from onset to puncture	0.88 (0.62–1.26)	0.496		
Time from onset to recanalization	0.94 (0.66–1.32)	0.711		
Procedure time	1.31 (0.80–2.14)	0.287		

*AIS, acute ischemic stroke; OR, odds ratio; CI, confidence interval; TIA, transient ischemic attack; SBP, systolic blood pressure; DBP, diastolic blood pressure; LDL-C, low density lipoprotein cholesterol; NIHSS, National Institute of Health Stroke Scale; ASPECTS, Alberta Stroke Program Early CT Score; DSA, digital subtraction angiography; ASITN/SIR, American Society of Intervention and Therapeutic Neuroradiology/Society of Interventional Radiology; AOL, arterial occlusive lesion*.

* *Adjusted for sex, age, smoking, drinking, hypertension, diabetes, history of stroke or TIA, coronary heart disease, atrial fibrillation, and TOAST*.

### Predictors of Futile Recanalization in AIS Patients Treated With Mechanical Thrombectomy

**Figure 1:**
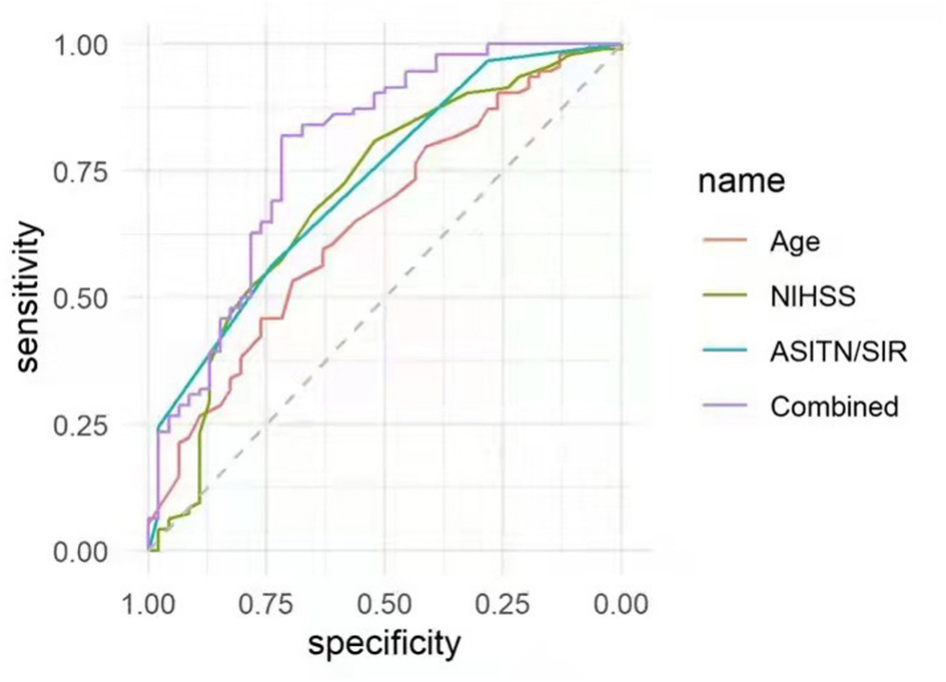
ROC curves were applied to analyze the efficacy of age, preoperative NIHSS score, and ASITN/SIR grade solely, or in combination, for prediction of futile recanalization in AIS patients treated with mechanical thrombectomy. NIHSS, National Institute of Health Stroke Scale; ASITN/SIR, American Society of Intervention and Therapeutic Neuroradiology/Society of Interventional Radiology; ROC, receiver operating characteristic; AIS, acute ischemic stroke.

Using the ROC curves from the above logistic regression analyses in futile recanalization, we evaluated the predictive efficacy of age, preoperative NIHSS score, and DSA ASITN/SIR grade. We found that the area under curve (AUC) for age was 0.65 (95% CI: 0.55–0.75), and the cut-off value was ≥71 years old (specificity 69.6%, and sensitivity 53.2%). The AUC for baseline NIHSS was 0.70 (95% CI: 0.60–0.80), and the cut-off value was ≥12 (specificity 52.2%, and sensitivity 80.9%). The AUC for ASITN/SIR grade was 0.73 (95% CI: 0.64–0.81), and the cut-off value was ≤ 3 (specificity 73.9%, and sensitivity 56.4%). Furthermore, the combination of age, NIHSS score, and ASITN/SIR grade had the highest AUC (0.75; 95% CI: 0.65–0.84), the specificity was 67.4%, and the sensitivity was 79.8% (Figure 1).

### ANA Score by Combined Parameter Analyses for Evaluating the Risk for Futile Recanalization in AIS Patients

Based on the above results, we integrated age, baseline NIHSS score, and collateral circulation ASITN/SIR grade, and constructed a prognosis after mechanical thrombectomy for AIS patients. Then the scoring index, ANA (Age-NIHSS-ASITN/SIR), was used to evaluate the risk for futile recanalization in those patients (Table 4).

**Table 4:** ANA score system derived from a combination of age, preoperative NIHSS score and ASITN/SIR grade in AIS patients with mechanical thrombectomy.

	**Cutoff-point**	**Score**
Age, year	<71	0
	≥71	1
Baseline NIHSS	<12	0
	≥12	1
DSA ASITN/SIR grade	>3	0
	≤ 3	1
ANA score	0–3

*ANA, Age-NIHSS-ASITN/SIR; NIHSS, National Institute of Health Stroke Scale; ASITN/SIR, American Society of Intervention and Therapeutic Neuroradiology/Society of Interventional Radiology; AIS, acute ischemic stroke; DSA, digital subtraction angiography*.

**Figure 2:**
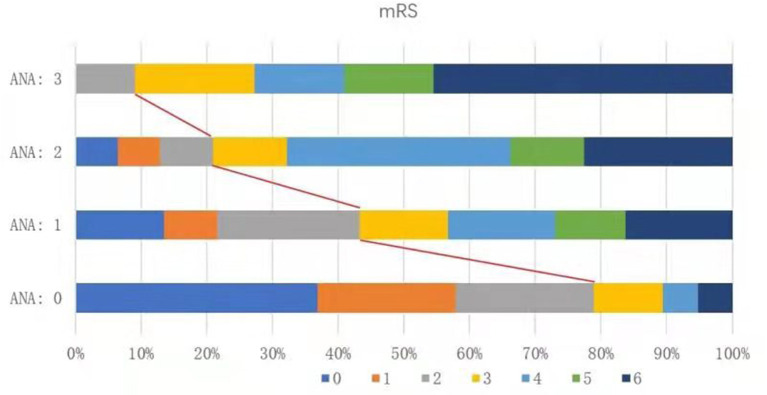
Proportion of different functional outcomes by mRS at 90 days post-operation among groups with different ANA scores in AIS patients treated with mechanical thrombectomy. mRS, modified Rankin Scale; ANA, Age-NIHSS-ASITN/SIR; AIS, acute ischemic stroke.

We divided the 140 patients into four groups according to the ANA score 0–3, and compared the distribution of 90-day mRS scores in these groups. Patients in the ANA score 0 group had the lowest rate of futile recanalization (mRS > 2), which was 21.05%, while patients in the ANA score 3 group had 90-day mRS > 2, which accounted for the highest rate (90.91%) and risk (OR: 50.07, 95% CI: 7.74–495.93) of futile recanalization (Figure 2; Table 5).

**Table 5:** Analyses of ANA score for the risk for futile recanalization in AIS patients treated with mechanical thrombectomy.

**ANA score**	**Total *N***	**Futile recanalization *n* (%)**	OR (95% CI)^*^	***P*-values**
0	19	4 (21.05%)	1 (Reference)	–
1	37	21 (56.76%)	6.32 (1.67–28.72)	0.01
2	62	49 (79.03%)	18.55 (5.03–83.65)	<0.001
3	22	20 (90.91%)	50.07 (7.74–495.93)	<0.001

*ANA, Age-NIHSS-ASITN/SIR; AIS, acute ischemic stroke; OR, odds ratio; CI, confidence interval*.

* *Adjusted for sex, age, smoking, drinking, hypertension, diabetes, history of stroke or TIA, coronary heart disease, atrial fibrillation, and TOAST*.

**Figure 3:**
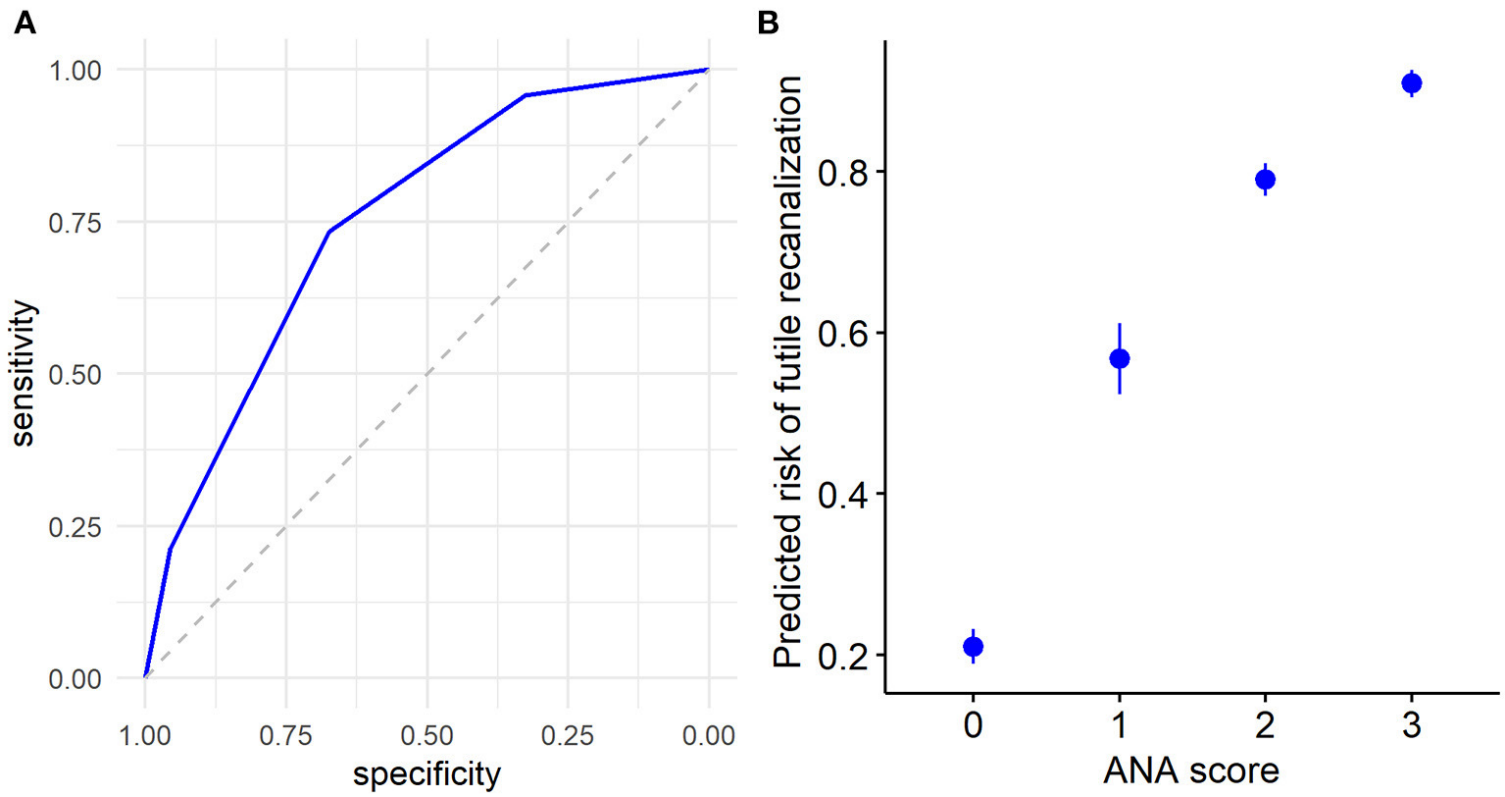
The predicted efficacy of ANA score for futile recanalization in AIS patients treated with mechanical thrombectomy. **(A)** The ROC curve was applied to analyze the efficacy of ANA score in prediction of futile recanalization. **(B)** Predicted probability 95% CI of ANA score for futile recanalization in AIS patients. ANA, Age-NIHSS-ASITN/SIR; AIS, acute ischemic stroke; ROC, receiver operating characteristic; CI, confidence interval.

Furthermore, we used the ROC curve analyses of ANA score to evaluate the predictive efficacy of futile recanalization in AIS patients. The AUC was 0.75 (95% CI: 0.67–0.83), the specificity was 67.4%, and the sensitivity was 73.4% (Figure 3A). Among the four ANA score groups, ANA = 0 group predicted the lowest risk for futile recanalization, and ANA = 3 group predicted the highest risk for futile recanalization (Figure 3B).

**Figure 4:**
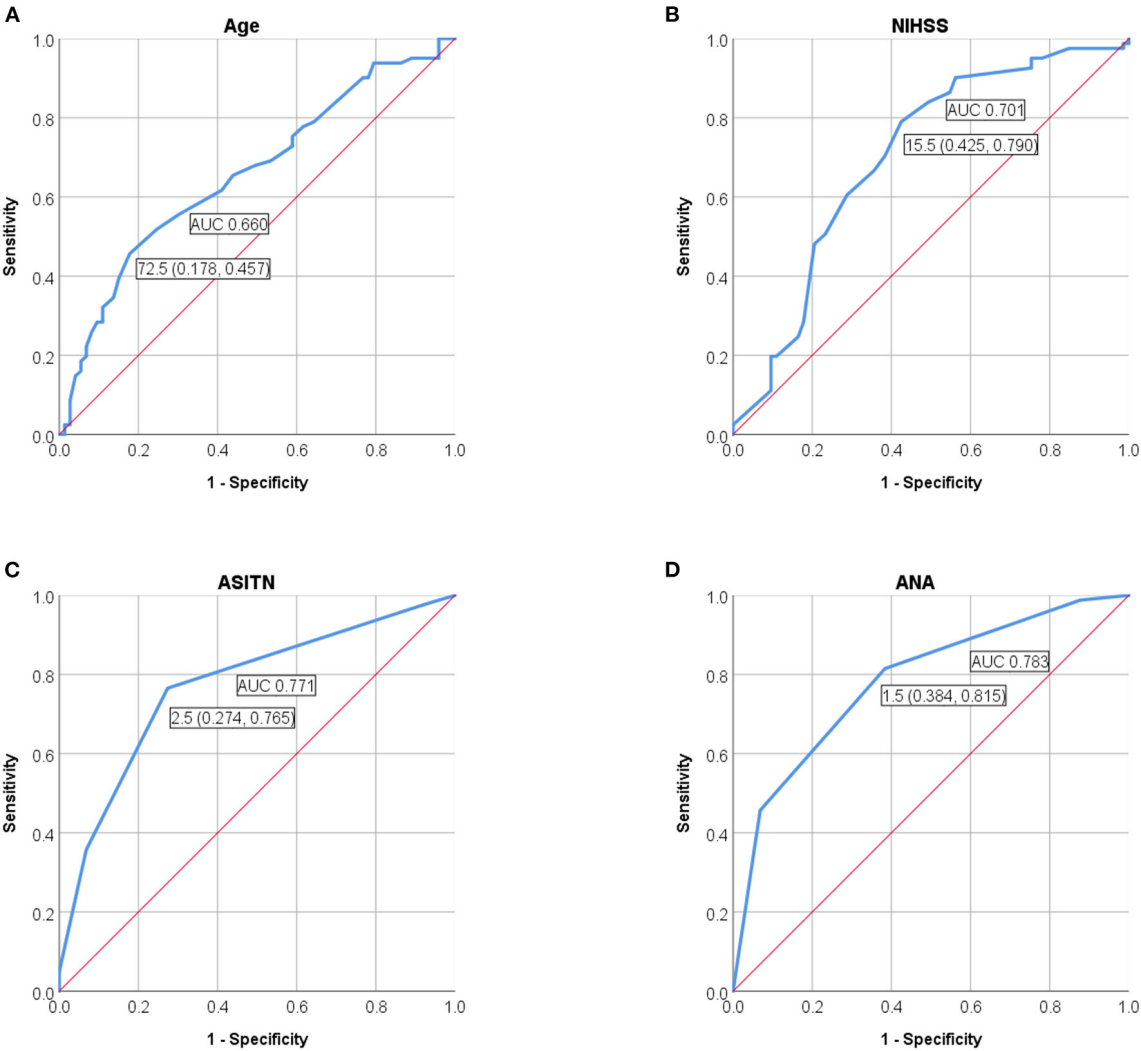
ROC curves were applied to analyze the efficacy of **(A)** age, **(B)** preoperative NIHSS score, and **(C)** ASITN/SIR grade solely, or **(D)** ANA score, for prediction of futile recanalization in AIS patients with thrombectomy on the validation cohort. NIHSS, National Institute of Health Stroke Scale; ASITN/SIR, American Society of Intervention and Therapeutic Neuroradiology/Society of Interventional Radiology; ANA, Age-NIHSS-ASITN/SIR; AUC, area under curve; ROC, receiver operating characteristic; AIS, acute ischemic stroke.

We also performed the ROC curve analyses of ANA score to evaluate the performance of prediction model for futile recanalization on the validation cohort. The AUC was 0.783 (95% CI: 0.711–0.855), the specificity was 61.6%, and the sensitivity was 81.5% (Figure 4).

## Discussion

We explored the risk factors for futile recanalization in AIS patients treated with mechanical thrombectomy. In addition, we constructed a novel scoring system for predicting this risk by integrating age, baseline NIHSS score, and DSA collateral circulation ASITN/SIR grade. We were also able to effectively predict the risk for futile recanalization by using the scoring system on the validation cohort. The combination of these three independent factors increased the complementary value for prediction of futile recanalization in these patients.

Despite progress in intravascular interventional techniques in recent years, futile recanalization continues to be a serious clinical problem during the implementation of mechanical thrombectomy. In our cohort, we demonstrated that the rate of meaningful recanalization was only 32.9%, while the rate of futile recanalization was 67.1% in AIS patients after mechanical thrombus removal and recanalization. By contrast, some international randomized controlled trials (RCTs) for endovascular treatment of AIS patients have reported that the proportion of meaningful recanalization is about 47.5% with strict screening of patients before operation (27–29). Therefore, the development of patients' screening criteria for mechanical thrombectomy is expected to be of great clinical significance for the improvement of meaningful recanalization.

A reliable predictor for futile recanalization is necessary to identify patients who would not benefit from mechanical recanalization. Our study demonstrated that the risk for futile recanalization was higher in our AIS cohort with NIHSS scores ≥ 12. It is generally believed that the most effective tool for predicting patients with large vessel occlusion is the NIHSS score after the initial diagnosis of AIS by neurologists and emergency physicians (30, 31). In a meta-analysis, it was found that the best balance of sensitivity and specificity was achieved when the NIHSS score was ≥10 to predict vascular occlusion (32). High baseline NIHSS score (NIHSS score > 10), older age (age > 70 years old), and delay in treatment were predictors of poor prognosis after complete recanalization (13, 14). Similarly, data from our patients older than 71 years old were at increased risk for futile recanalization.

In other studies, imaging parameters based on CT, DSA, and MRI have been tested and used either alone or in combination to select patients suitable for mechanical thrombectomy, and to improve the probability of meaningful recanalization (6, 33). A series of RCT studies have reported satisfactory positive results using strict imaging indicators to screen suitable patients (22, 34). A number of studies have also shown that collateral flow assessed by DSA can predict response to endovascular therapy for AIS and the status of angiographic collateral blood flow can assist in the decision-making of mechanical thrombectomy treatment (35). DSA has become the gold standard for evaluating cerebral collateral blood flow (36). However, there is no research on collateral flow as a predictor for futile recanalization after endovascular treatment until now. Here, we aimed to determine the predictive value of real status of cerebral collateral blood flow in futile recanalization. In our study, we found that patients with a good collateral circulation state (ASITN/SIR grade > 3) before endovascular treatment had a low risk for futile recanalization. Studies have reported that good collateral circulation state is significantly related to the final smaller infarct core volume, lower hemorrhage transformation rate, and lower mortality (37). However, evaluation of ASITN/SIR grade depends on the proficiency and experience of the technical operator. In recent years, with the development of non-invasive angiography technology, some studies have adopted methods including multiphase CT perfusion (MCTP) and dynamic CTA to evaluate the collateral circulation status of patients with AIS (38–40). Therefore, further study will focus on collateral status grading performed on CTA prior to thrombectomy, and thereby a double-check made by DSA, which could have more sense in the development of a reliable score predicting futile recanalization related to the appropriateness and effectiveness of thrombectomy.

In this study, patients in the ANA score 0 group had the lowest rate of futile recanalization (21.05%), while patients in the ANA groups 2 and 3 had the high risk of futile recanalization (79.03 and 90.91%, respectively). Therefore, according to the ANA score 2–3 in the present study, these patients may be excluded from the decision-making of thrombectomy treatment although they fulfill the necessary guideline criteria (24). However, in this study, the relatively low AUC, specificity and sensitivity of ANA score were detected, which may be due to the small sample, especially in the division of 140 patients into 4 groups and the further reduced number of patients per group. Secondly, for the thrombectomy performed between 6 and 24 h from symptom onset in the present study, the different inclusion criteria had been adopted (17, 23, 24). Furthermore, previous studies reported that female gender and delayed endovascular treatment were also regarded as the independent predictors of unfavorable outcomes in recanalized patients, besides age and NIHSS score (7, 8). Thus, further studies with a larger sample size are needed to perform subgroup analysis according to the female gender and procedural time, and to adopt the consistent mismatch criteria between clinical deficit and infarct volume according to the DAWN trial for the late window patients (17), which may contribute to increasing both specificity and sensitivity of ANA score and aid in screening suitable patients for meaningful recanalization much more accurately.

This study was based on actual clinical data. As a retrospective cohort study based on participants from 3 centers, we acknowledged that there were certain limitations. First, the sample size was relatively small. Second, evaluation of the collateral circulation by ASITN/SIR was derived from the invasive DSA examination, which may limit its preoperative application before mechanical thrombectomy. In addition, the relatively low specificity and sensitivity of ANA score, and the different inclusion criteria for the thrombectomy performed beyond 6 h from symptom onset were shown in this study. At present, there is still a lack of Chinese AIS data on prognosis of thrombectomy in the clinical guidelines, and future researches will be necessary to validate our conclusions by prospective cohort or RCT studies in larger sample of patients.

## Conclusions

We demonstrate that older age, higher baseline NIHSS score, and lower ASITN/SIR grade are independent risk factors for futile recanalization in AIS patients. The ANA score, which combines patient age, NIHSS score, and ASITN/SIR grade, can effectively predict the risk for futile recanalization. This score may be helpful for selecting potential AIS patients before conducting mechanical thrombectomy. Further studies are necessary to validate the prognostic value of this combined score solely and in combination with other clinical factors for futile recanalization.

## Data Availability Statement

The raw data supporting the conclusions of this article will be made available by the authors, without undue reservation.

## Ethics Statement

The studies involving human participants were reviewed and approved by Independent Ethics Committee of Shanghai Ninth Hospital, Shanghai Jiao Tong University School of Medicine. Written informed consent for participation was not required for this study in accordance with the national legislation and the institutional requirements.

## Author Contributions

JS designed the study and helped revise this manuscript. JL conceived the study and made final approval of this manuscript. HP and CL analyzed all data and prepared the drafting of this article. LC, YQ, and PH contributed to the acquisition of clinical data. BL and YZ provided the validation cohort. All authors contributed to the article and approved the submitted version.

## Conflict of Interest

The authors declare that the research was conducted in the absence of any commercial or financial relationships that could be construed as a potential conflict of interest.

## Publisher's Note

All claims expressed in this article are solely those of the authors and do not necessarily represent those of their affiliated organizations, or those of the publisher, the editors and the reviewers. Any product that may be evaluated in this article, or claim that may be made by its manufacturer, is not guaranteed or endorsed by the publisher.
